# A Hybrid Circuit for Spoof Surface Plasmons and Spatial Waveguide Modes to Reach Controllable Band-Pass Filters

**DOI:** 10.1038/srep16531

**Published:** 2015-11-10

**Authors:** Qian Zhang, Hao Chi Zhang, Han Wu, Tie Jun Cui

**Affiliations:** 1 State Key Laboratory of Millimeter Waves, Southeast University, Nanjing 210096, China; 2 Synergetic Innovation Center of Wireless Communication Technology, Southeast University, Nanjing 210096, China; 3 Cooperative Innovation Centre of Terahertz Science, No. 4, Section 2, North Jianshe Road, Chengdu 610054, China

## Abstract

We propose a hybrid circuit for spoof surface plasmon polaritons (SPPs) and spatial waveguide modes to develop new microwave devices. The hybrid circuit includes a spoof SPP waveguide made of two anti-symmetric corrugated metallic strips and a traditional substrate integrated waveguide (SIW). From dispersion relations, we show that the electromagnetic waves only can propagate through the hybrid circuit when the operating frequency is less than the cut-off frequency of the SPP waveguide and greater than the cut-off frequency of SIW, generating efficient band-pass filters. We demonstrate that the pass band is controllable in a large range by designing the geometrical parameters of SPP waveguide and SIW. Full-wave simulations are provided to show the large adjustability of filters, including ultra wideband and narrowband filters. We fabricate a sample of the new hybrid device in the microwave frequencies, and measurement results have excellent agreements to numerical simulations, demonstrating excellent filtering characteristics such as low loss, high efficiency, and good square ratio. The proposed hybrid circuit gives important potential to accelerate the development of plasmonic integrated functional devices and circuits in both microwave and terahertz frequencies.

Surface plasmon polaritons (SPPs) are a kind of propagating surface waves bounded on the interface of metal and dielectric at the optical frequency^1^. In virtue of strong local-field enhancement and the diffraction limit broken, SPPs have attracted many scientists to make investigations^2^. The plasmonic waves propagate in directions parallel to the surface of metal and decay exponentially normal to the surface^3^,^4^. Different from metal with the characteristic of negative permittivity at the optical frequency, the metal behaves like a perfectly electric conductor in the terahertz and microwave frequencies where we cannot find SPPs tightly confined as a result^5^. Therefore, the spoof SPP metamaterials^6^,^7^ have been proposed to obtain the plasmon propagation at such frequencies. The metamaterials of corrugated metal structures^7^,^8^,^9^,^10^,^11^,^12^,^13^ such as one dimensional or two dimensional periodic arrays enchased with slits, holes or blocks have been employed to support the spoof SPP modes, which have the same dispersion relations and spatial confinements with SPPs in the optical region. For facilitating the application of spoof SPPs, broadband and high-efficiency conversion and transition^14^,^15^ between traditional transmission line and conformal SPPs with double grating and single grating have been reported, which allow for successful combination between the spatial guided waves and SPPs. At the same time, some devices based on spoof SPPs have been developed, including ultra-wideband surface plasmonic filter^16^, efficient converter^17^,^18^ between SPP modes and spatial radiated modes, and controlling rejecter of SPPs^19^ and so on^20^,^21^,^22^,^23^,^24^. All of the reported papers attest that the spoof SPPs can provide the advantage of miniaturization and localized electromagnetic (EM) waves in subwavelength scales.

The substrate integrated waveguide (SIW) and similar schemes^25^,^26^,^27^,^28^,^29^ have been put forward in recent years as new spatial guided-wave structures, which can be integrated in the dielectric substrate with the characteristics of low insertion loss and low radiation. Compared with the traditional expensive and cumbersome waveguide, SIWs have similar performance but more compact dimensions, which makes it possible to downsize the microwave system into a small package.

In this article, we propose a hybrid circuit of the spoof SPP waveguide which supports SPP modes and SIW that is a barrier of spatial guided modes. The spoof SPP waveguide takes the advantage of the anti-symmetrical corrugated metallic strips^22^ which bring further tighter EM field confinement and smaller propagating wavelength for decreasing the interference and miniaturizing the system at the same frequency. Linear tapered microstrip lines^27^,^30^ serve as a bridge to connect the SPP waveguide, SIW and microstrip line, attaining the conversions among the transverse magnetic (TM) mode, transverse electric (TE) mode, and quasi-transverse-electromagnetic (TEM) mode. Numerical and experimental resulted are present to validate the new features of the hybrid circuit.

## Results

### Hybrid circuit of spoof SPP waveguide and SIW

**Figure 1:**
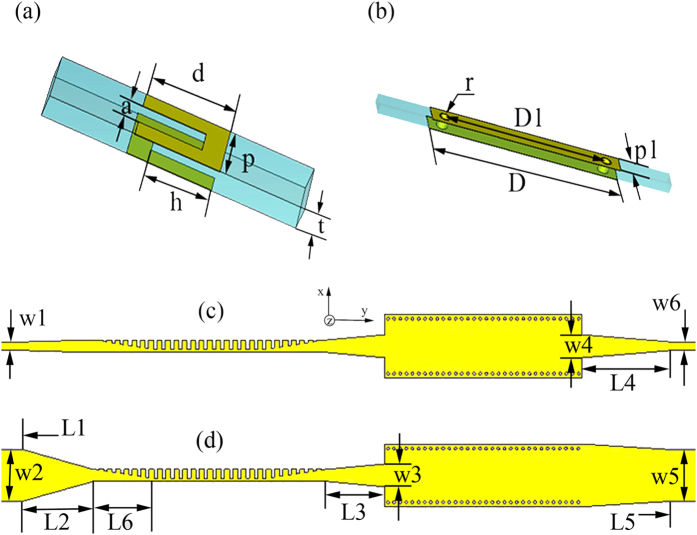
Schematic pictures of the hybrid circuit containing the spoof SPP waveguide and SIW, which is divided into two main sections. (**a**) A unit cell of the spoof SPP waveguide section. (**b**) A unit cell of the SIW section. (**c**) The top view of the hybrid circuit, including the connection microstrip lines. (**d**) The bottom view of the hybrid circuit. All insets show the detailed parameters.

We propose a hybrid circuit that contains a spoof SPP waveguide, SIW, and tapered microstrip lines, as illustrated in Fig. 1. The hybrid circuit is divided into two main sections of SPP waveguide and SIW. The commercial printed circuit board, F4B^31^, is used as the dielectric substrate with thickness of *t* = 0.5 mm and relative permittivity of 2.65. We choose annealed copper (electric conductivity *σ* = 5.8e + 007 S/m) as metal layers whose thickness is 0.018 mm. The first main section of the hybrid circuit is the spoof SPP waveguide, which is composed of two anti-symmetrical corrugated metallic strips^22^. Figure 1(a) shows a unit cell of the SPP waveguide, where *a* and *h* denote the groove width and depth, and *d* is strip width, respectively. The SPP waveguide is formed by arranging such unit along the *x*-axis with period *p* on the top and bottom surfaces of the dielectric substrate.

In the second main section, we adopt a typical configuration of SIW^32^, whose unit cell is depicted in Fig. 1(b). The upper and lower surfaces of the dielectric substrate are metal layers, and the dielectric substrate is separated by two rows of metallic via holes which are separated by a certain distance *D1*. The period of via holes and via diameter are donated as *p1* and *r*, respectively.

The two main sections are connected by tapered microstrip lines. There are a variety of tapered microstrip-line forms, such as arc taper lines and linear taper lines. Here, we choose the linear tapered microstrip lines^27^. Figure 1(c,d) illustrate three tapered microstrip lines, including the connection between 50 Ω-microstrip line and the SPP waveguide, the connection between SPP waveguide and SIW, along with the connection between SIW and 50 Ω-microstrip line. In such figures, *L2, L3, L4* and *w1, w2, w3, w4, w5, w6* represent the lengths and widths of such three tapers, respectively. The first taper (part *L2* and *L6*) is used to transform the quasi-TEM mode of the microstrip line into the TM mode of the SPP waveguide, the second taper (part *L3*) is for the transformation from the TM mode to the TE_10_ mode, and the last taper (part *L4*) transforms the TE_10_ mode to the quasi-TEM mode.

We employ the eigen-mode solver of the commercial software, CST Microwave Studio, to calculate the dispersion relations of the SPP waveguide and comprehending propagation characteristics of the waveguide. In CST Microwave Studio, we set the boundary condition as periodic in the *x* axis, and electric (Et = 0) in both *y* and *z* axes. Owing to the physical intermodal coupling for the forward mode and backward mode, there will be totally reflection of the incident energy when the working frequency is upper than the cutoff frequency^33^. That is to say, while the input frequencies outweigh the cutoff frequency, the propagating wave vectors of the forward and back-forward modes have the opposite directions but the same value, which result in the zero transmission.

It is rather difficult to analyze the hybrid circuit directly. Considering the fact that SIW is composed of two metal surfaces and two rows of metallic via holes to approximate a kind of rectangular waveguide, we can establish the corresponding relationship between the traditional rectangular waveguide and SIW. Then the design of SIW devices can be changes as the design of traditional rectangular waveguide devices, which thereby greatly reduces the complexity and design time. Thus, in ref. 32, the authors made use of accurate results obtained from the method of lines to derive an empirical equation for the normalized width of SIW, which is convenient and handy for design^32^:


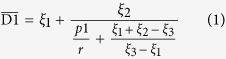


where


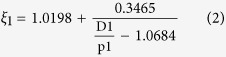



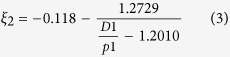



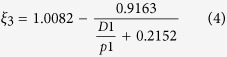


in which 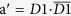 is the equivalent width of SIW. From the formula, the corresponding equivalent waveguide width can be straightly launched from the structural parameters (*D1*, *p1* and *r*) of the designed SIW. The phase characteristics can be obtained from the theoretical analysis of the equivalent metal waveguide. Meanwhile, the dispersion relationship of SIW and the light line in the dielectric substrate are calculated analytically as


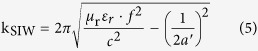



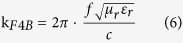


**Figure 2:**
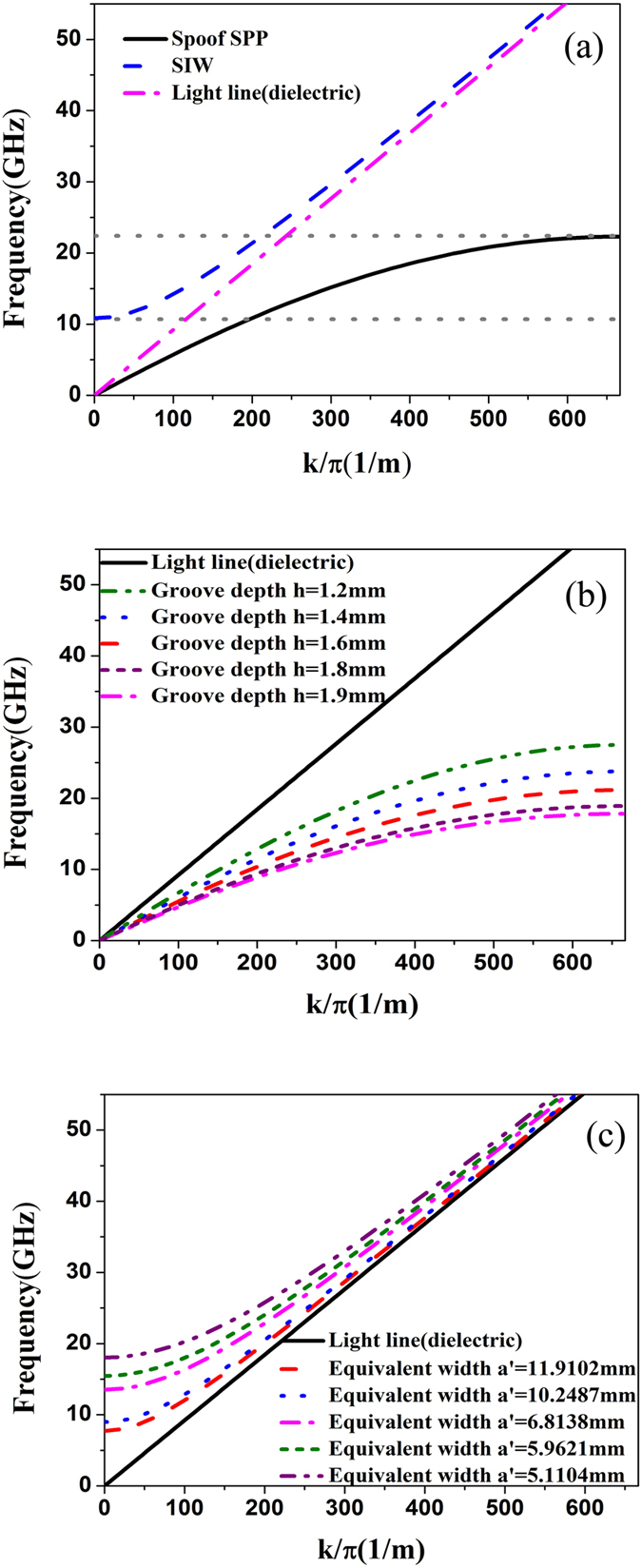
(**a**) Dispersion diagrams of the SPP waveguide, SIW, and light line in the dielectric substrate with the dielectric constant of 2.65 and loss tangent of 0.001. (**b**) Dispersion diagrams of SPP waveguides with different groove depths, in which *h* = 1.2 mm, 1.4 mm, 1.6 mm, 1.8 mm and 1.9 mm. (**c**) Dispersion diagrams of SIWs with different equivalent widths, in which a′ = 11.9102 mm, 10.2487 mm, 6.8138 mm, 5.9621 mm and 5.1104 mm.

Then the dispersion curves of the designed SPP waveguide and SIW, and the light line in dielectric substrate are illustrated in Fig. 2(a), as marked by black, blue, and magenta lines, respectively. Here, the geometrical parameters are chosen as *p* = 1.5 mm, *a* = 0.6 mm, *h* = 1.5 mm, *d* = 2 mm; *D* = 10.31 mm, *r* = 0.5 mm, *D1* = 8.81 mm, and *p1* = 1.1 mm, acquiring the equivalent width a′ = 8.5173 mm. From the blue and black curves, we notice that there is a significant transmission passband between the cutoff frequencies of SPP waveguide and SIW for the designed geometrical parameters.

In Fig. 2(b), we clearly observe that the dispersion curve of SPP waveguide deviates gradually from the light line when k/*π* grows up, and that the curves asymptotically approach to different cutoff frequencies as the groove depth *h* increases from 1.0 to 1.9 mm. Consequently, on the basis of different groove depths, we can achieve various dispersion curves and propagating vectors on the SPP waveguides. However, the dispersion curve of the spoof SPP has a tendency to the light line by decreasing the groove depth. Figure 2(c) shows dispersion curves of SIW, which is gradually close to the light line. It is obvious that the propagation constant *k* is greater than zero when the frequencies are greater than the cutoff frequency. When the equivalent width are chosen as 11.9102 mm (*D* = 14.42 mm, *p1* = 1.54 mm, *r* = 0.7 mm and *D1* = 12.32 mm), 10.2487 mm (*D* = 12.40 mm, *p1* = 1.32 mm, *r* = 0.6 mm and *D1* = 10.6 mm), 6.8138 mm (*D* = 8.248 mm, *p1* = 0.88 mm, *r* = 0.4 mm and *D1* = 7.048 mm) and 5.1104 mm (*D* = 6.186 mm, *p1* = 0.66 mm, *r* = 0.3 mm and *D1* = 5.286 mm), we notice that the cutoff frequency of SIW increases with the decrease of the equivalent width. For the tunable performance of the spoof SPPs and SIW, we can realize expediently controls of upper and lower cutoffs of the passband, generating controllable band-pass filters.

### Numerical simulations and physical interpretations

**Figure 3:**
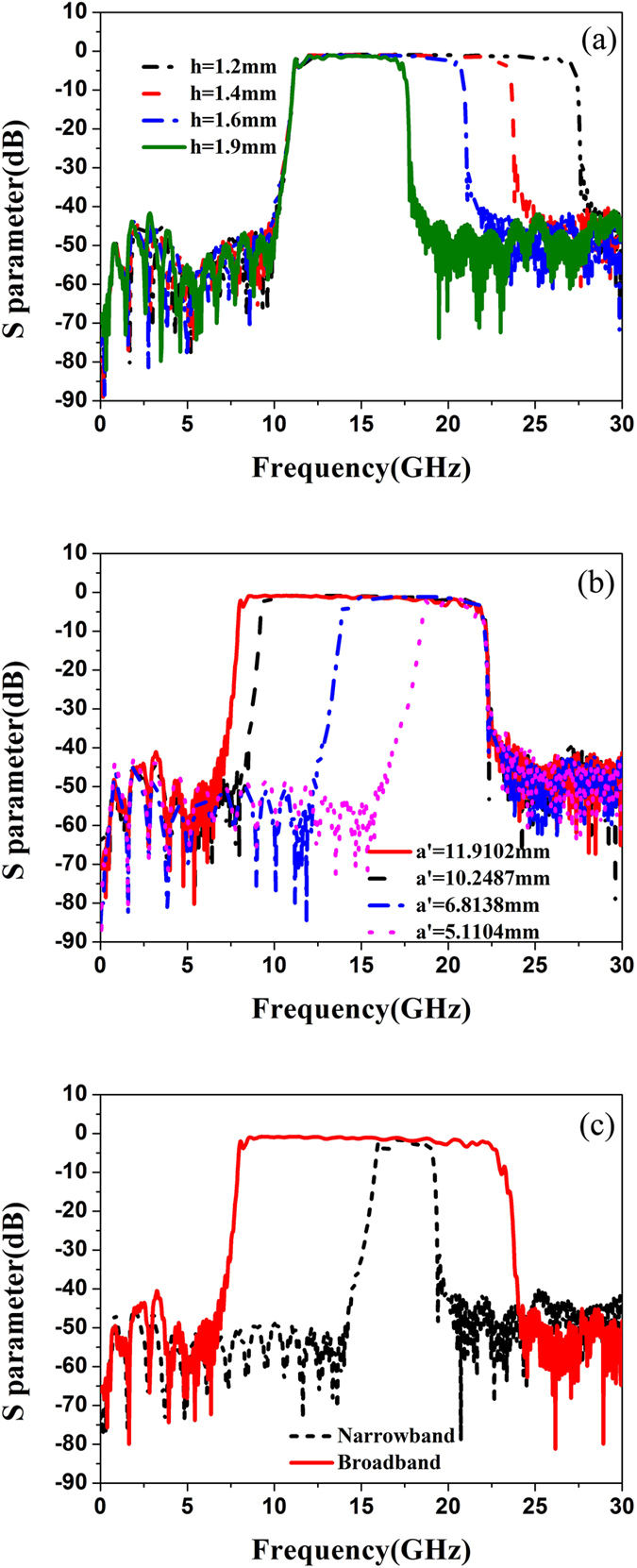
(**a**) The simulated transmission coefficients of the SPP-SIW hybrid circuit with the equivalent width a′ = 8.5173 mm and different groove depths *h* = 1 mm, 1.2 mm, 1.4 mm, and 1.8 mm. (**b**) The simulated transmission coefficients of the SPP-SIW hybrid circuit with the groove depth h = 1.5 mm and different equivalent widths a′ = 11.9102 mm, 10.2487 mm, 6.8138 mm and 5.11024 mm. (**c**) In view of different groove depths and equivalent widths, the designed ultra-wideband transmission ranging from 8.01 to 22.41 GHz with *h* = 1.4 mm and a′ = 11.9102 mm, and the narrow band transmission ranging from 16.11 to 18.00 GHz with *h* = 1.8 mm and 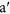 = 5.9621 mm.

To better understand the influence of groove depth *h* and equivalent width a′ on the transmission coefficient, we design the SPP-SIW hybrid circuits with different parameters. The simulated transmission coefficients of the hybrid circuit by the time-domain solver in CST Microwave Studio are demonstrated in Fig. 3(a), in which we set boundary conditions open spaces in all directions. We easily discover that the groove depth can control independently the upper cutoff frequency of the pass band but has little influence on the lower cutoff frequency, where *L1* = 4.0373 mm, *L2* = 12.5854 mm, *L3 = *10.3163 mm, *L4 = *15.9112 mm, *L5* = 4.8488 mm, *L6* = 10.3605 mm, a′ = 8.5173 mm, and *h* ranges from 1 mm to 1.9 mm. For the black (*h* = 1.2 mm), red (*h* = 1.4 mm), blue (*h* = 1.6 mm) and green (*h* = 1.9 mm) curves, their −3 dB bandwidths are 15.84 GHz, 11.73 GHz, 9.39 GHz and 5.91 GHz, respectively. Corresponding to the above curves, their highest transmission coefficients are −0.82 dB, −0.86 dB, −0.95 dB and −1.1 dB, respectively. Figure 3(b) depicts that the equivalent width of SIW can control the lower cutoff frequency of the pass band but has little influence on the upper cutoff frequency, in which most of the parameters have the same values as above except that *h* = 1.5 mm and a′ranges from 11.9102 mm to 5.11024 mm. Corresponding to the red, black, blue and magenta curves, The −3 dB bandwidths are 13.57 GHz, 12.54 GHz, 7.7 GHz, and 2.28 GHz, respectively, with the highest transmission coefficients as −0.83 dB, −0.86 dB, −1.1 dB, and −1.5 dB. Hence, it is convenient to control the upper and lower cutoff frequencies by adjusting the groove depth and equivalent width with lower loss.

Based on these characteristics, we design an ultra-wide pass-band filter and a narrow pass-band filter, whose simulated transmission coefficients are exhibited in Fig. 3(c). The ultra-wideband filter has the −3 dB bandwidth of 14.4 GHz (from 8.01 to 22.41 GHz) and the highest transmission coefficient of −0.81 dB with *h* = 1.4 mm and a′ = 11.9102 mm; while the narrow-band filter has the −3 dB bandwidth of 1.89 GHz (from 16.11 to 18.00 GHz) and the highest transmission coefficient of −1.45 dB with *h* = 1.8 mm and a′ = 5.9621 mm (*D* = 7.217 mm, *p1* = 0.77 mm, *r* = 0.35 mm and *D1* = mm). Certainly, all the transmission coefficients shown in Fig. 3 have excellent filtering characteristics with minimum insertion loss less than −1.5 dB. It is well known in the microwave community that the designs of the ultra-wideband filter and narrow-band filters are big challenges by using the conventional filter synthesize methods, such as the LC filter circuits and guided-wave circuits. The proposed hybrid SPP-SIW circuit solves the problems. Most importantly, compared with the previous ultra-wideband filters based on either SPP^16^ or SIW^34^,^35^, the main physical significance of this device is the successful conversion between the SPP modes and spatial-waveguide modes in either wideband or narrowband, which is controllable by a simple parameter.

**Figure 4:**
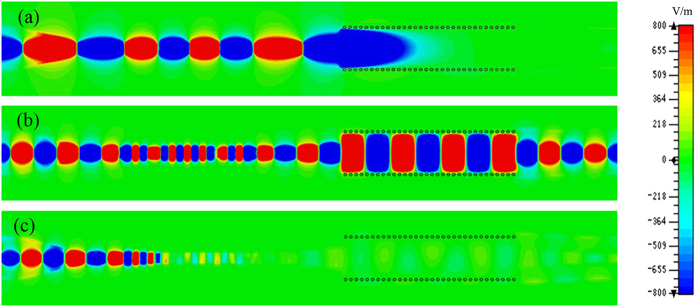
The simulated near electric-field distributions on the *x-y* plane that is inside the dielectric substrate at (**a**) 9 GHz (the lower frequency stopband), (**b**) 16 GHz (the passband), (**c**) 20 GHz (the upper frequency stopband), respectively.

To get a physical insight into the SPP-SIW hybrid circuit, we discuss the near electric fields (*z*-components) on the *x*–*y* plane, which is in the central plane inside the dielectric substrate. In CST Microwave Studio, we set up a field monitor for electric fields at a few frequencies. We take a sample waveguide with the parameters chosen in Fig. 2(a), and the simulated field distributions are illustrated in Fig. 4(a,c) at the lower frequency stopband and upper frequency stopband, where there are no fields at 9 GHz in SIW and at 20 GHz in SPP waveguide, respectively. At 9 GHz of the lower stopband, the waves stop propagating in the beginning of SIW; at 20 GHz of the upper stopband, the waves are cutoff on the SPP waveguide after the matching section. However, within the pass band, the waves are propagating through the SPP waveguide and SIW with very small loss, as shown in Fig. 4(b). We notice that the tapered microstrip lines can effectively realize the coupling and conversion among different propagating waves in the hybrid circuit. This provides an intuitionistic proof to corroborate the transmission waveguide filtering performance.

### Measurement results and discussions

**Figure 5:**
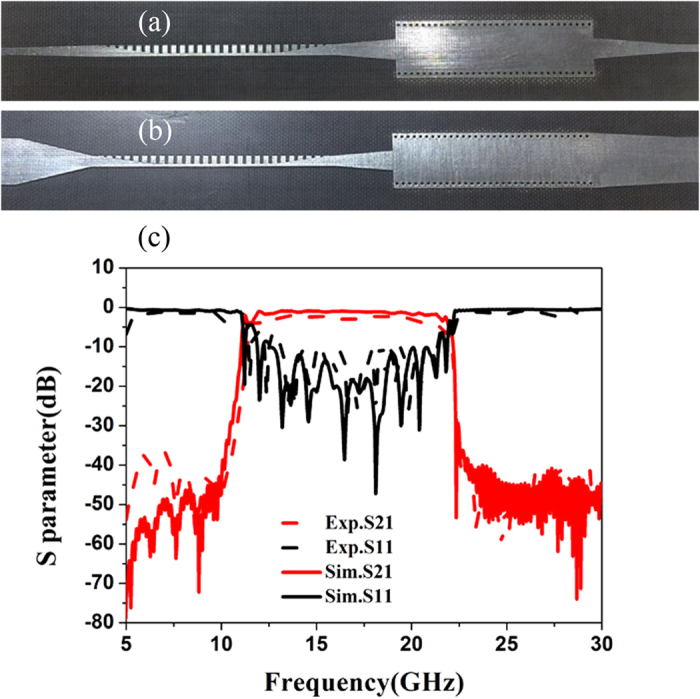
(**a**,**b**) The photographs of the front and back surfaces of the SPP-SIW hybrid circuit. (**c**) The measured and simulated transmission coefficients (S_21_) and reflection coefficients (S_11_) of the fabricated sample.

To further study the operation performance quantitatively of the SPP-SIW hybrid circuit, we fabricate a sample using the F4B substrate, as exhibited in Fig. 5(a,b) for the front and back views which are photographed by Qian Zhang. The length and width of the fabricated sample are 124.899 and 20 mm, respectively. The groove depths *h* = 1.5 mm and equivalent width a′ = 8.5173 mm are chosen in this experiment. We use two 50 Ω coaxial lines to connect the fabricated sample welded two standard SMA connectors and the Agilent vector network analyzer (VNA, N5230C). We have measured the S scattering parameters of the sample, as illustrated by the red and black dotted lines in Fig. 5(c). As comparisons, numerical simulations are also given in the same figure, as shown by the red and black solid lines. It is clearly observed that the simulated results have excellent agreements to the measured results. From the reflection coefficients (S_11_), we note that S_11_ is almost less than −10 dB in the whole frequency band from 11.92 to 21.54 GHz. From the transmission coefficients (S_21_), we notice that the proposed circuit has excellent filtering characteristics such as high transmission, good bandpass, and great square ration. The maximum transmission coefficient can reach −0.5 dB and the −3 dB bandwidth is as high as 9.62 GHz.

Regarding to the important parameter of a filter, the square ratio, the calculation formula is written as:


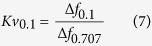


It is widely known that 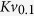 is closer to one, and the rectangle is more steep. This value is a very good proof of the quality of the square ratio. For the simulation results of this sample, the working frequencies of −20 dB are 10.86 GHz and 22.28 GHz. Hence we can obtain 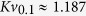. It is because of two points that the filter has such a good square ratio. Through analyzing dispersion curves and electric-field distributions, we unambiguously acquaint that the lower and upper cutoff frequencies are controlled by SIW and SPP waveguide, respectively. Hence, one point is based on SIW. Since we can get the SIW properties from theoretical analysis of equivalent metal waveguide, the TE_10_ mode of SIW is described by


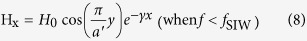


in which 
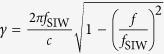
. We call it the decaying mode which is a rapidly attenuating oscillation mode. Hence there are no energy and power transmitting.

The other point is because of the spoof SPPs. When 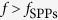, the energy and wave vectors of the forward modes are in the opposite direction from the backward modes. As a result, the propagation constants of the forward and backward modes become vestigial, leading to the total reflection. Then the propagation velocity becomes zero and there are no energy and power.

## Conclusion

Utilizing the traditional SIW and spoof SPP waveguide designed with antisymmetrical corrugated metallic strips, we have proposed a novel hybrid circuit to develop highly efficient and pass-band controllable filters. Owning to the highly confined EM waves of SPPs and lower radiation losses of SIW, the proposed hybrid circuit has excellent transmission efficiency and low loss. Meanwhile, the good square ratio benefits from the total reflections of SPPs and decaying modes of SIW. We have modulated the groove depth and equivalent width to design ultra wideband and narrowband filters, whose performances are splendid as well. To verify the marvelous capability, we have fabricated a sample using the commercial dielectric substrate, F4B. Both simulation and measurement results have demonstrated the highly efficient transmissions. The proposed SPP-SIW hybrid circuit and tunable passband filters provide potentials to advanced plasmonic functional devices and integrated circuits.

## Methods

**Figure 6:**
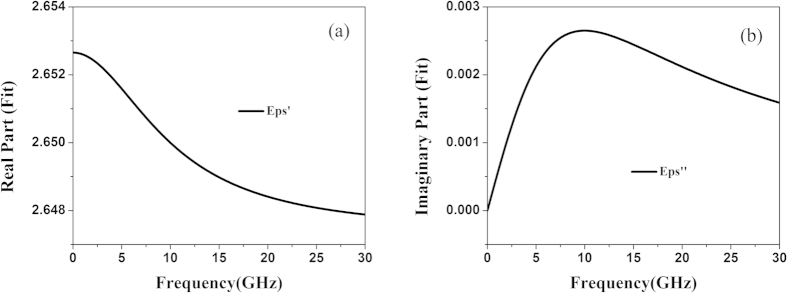
The F4B’s electric dispersion of the first-order model in the simulation band. (**a**) The real part of the dielectric constant. (**b**) The imaginary part of the dielectric constant.

All numerical simulations of SPP units, hybrid circuits and E-fields are performed by the commercial software, CST Microwave Studio, whose modeling circumstance has been explicated in details. F4B, a kind of Teflon woven, is used for the simulation model and experimental fabrication. Figure 6 shows the real part and imaginary part of the dielectric constant, respectively. It can be clearly observed that F4B is relatively stable in the simulation band, and its dispersion has little effect on the simulation and experiment results.

## Additional Information

**How to cite this article**: Zhang, Q. *et al.* A Hybrid Circuit for Spoof Surface Plasmons and Spatial Waveguide Modes to Reach Controllable Band-Pass Filters. *Sci. Rep.* **5**, 16531; doi: 10.1038/srep16531 (2015).
